# Clinical Simulation in the Regulation of Software as a Medical Device: An eDelphi Study

**DOI:** 10.2196/56241

**Published:** 2024-06-25

**Authors:** Fiona O'Driscoll, Niki O'Brien, Chaohui Guo, Matthew Prime, Ara Darzi, Saira Ghafur

**Affiliations:** 1 Institute of Global Health Innovation Imperial College London London United Kingdom; 2 Roche Diagnostics Basel Switzerland

**Keywords:** digital health technology, software as a medical device, clinical simulation, Delphi study, eDelphi study, artificial intelligence, digital health

## Abstract

**Background:**

Accelerated digitalization in the health sector requires the development of appropriate evaluation methods to ensure that digital health technologies (DHTs) are safe and effective. Software as a medical device (SaMD) is a commonly used DHT by clinicians to provide care to patients. Traditional research methods for evaluating health care products, such as randomized clinical trials, may not be suitable for DHTs, such as SaMD. However, evidence to show their safety and efficacy is needed by regulators before they can be used in practice. Clinical simulation can be used by researchers to test SaMD in an agile and low-cost way; yet, there is limited research on criteria to assess the robustness of simulations and, subsequently, their relevance for a regulatory decision.

**Objective:**

The objective of this study was to gain consensus on the criteria that should be used to assess clinical simulation from a regulatory perspective when it is used to generate evidence for SaMD.

**Methods:**

An eDelphi study approach was chosen to develop a set of criteria to assess clinical simulation when used to evaluate SaMD. Participants were recruited through purposive and snowball sampling based on their experience and knowledge in relevant sectors. They were guided through an initial scoping questionnaire with key themes identified from the literature to obtain a comprehensive list of criteria. Participants voted upon these criteria in 2 Delphi rounds, with criteria being excluded if consensus was not met. Participants were invited to add qualitative comments during rounds and qualitative analysis was performed on the comments gathered during the first round. Consensus was predefined by 2 criteria: if <10% of the panelists deemed the criteria as “not important” or “not important at all” and >60% “important” or “very important.”

**Results:**

In total, 33 international experts in the digital health field, including academics, regulators, policy makers, and industry representatives, completed both Delphi rounds, and 43 criteria gained consensus from the participants. The research team grouped these criteria into 7 domains—background and context, overall study design, study population, delivery of the simulation, fidelity, software and artificial intelligence, and study analysis. These 7 domains were formulated into the simulation for regulation of SaMD framework. There were key areas of concern identified by participants regarding the framework criteria, such as the importance of how simulation fidelity is achieved and reported and the avoidance of bias throughout all stages.

**Conclusions:**

This study proposes the simulation for regulation of SaMD framework, developed through an eDelphi consensus process, to evaluate clinical simulation when used to assess SaMD. Future research should prioritize the development of safe and effective SaMD, while implementing and refining the framework criteria to adapt to new challenges.

## Introduction

COVID-19 has accelerated digitization in the health sector and there is an urgent need for appropriate evaluation methods to ensure that digital health technologies (DHTs) are safe and effective. Software as a medical device (SaMD), defined by the International Medical Device Regulators Forum as “software intended to be used for one or more medical purposes that perform these purposes without being part of a hardware medical device” [1], is a DHT that is growing in relevance [2]. Yet, existing regulations on medical devices were developed before the widespread development of SaMD and are ill-suited for this type of DHT.

Discussions on common frameworks and principles for SaMD, including key definitions [1], framework for risk categorization [3], quality management system [4], and clinical evaluation [5], have left gaps. The latest literature on the regulation of SaMD calls for international standards and guiding principles addressing the unique iterative nature of SaMD [6,7]. Several examples outlining the challenges have recently emerged. In June 2023, iRhythm received a letter from the US Food and Drug Administration (FDA) outlining serious violations following challenges that were made to the SaMD’s hardware, firmware, and algorithm that potentially require new 510 (k) submissions [8,9]. The regulator’s concern was the potential impact of these changes on the safety or effectiveness of iRhythm. The development of a digital solution for multiple sclerosis highlighted the key considerations required for developing a safe and clinically validated SaMD, raising the topics of technical verification, clinical evaluation, cybersecurity, and data privacy [10]. A 2022 study conducted by Rock Health and John Hopkins University examining US-based digital health start-ups found that 44% of these companies had no published clinical trials or regulatory filings to support their digital health solutions [11]. Fundamentally, the challenge to SaMD developers is how to manage evidence generation to support the assertion that the device remains safe and effective as per regulatory requirements each time iterative changes are made to hardware and firmware.

Novel approaches to evidence generation, including real-world evidence and clinical simulation, are being developed to support the development of DHTs to market and maintaining regulatory adherence over time. Clinical simulation research puts the intended end users, such as clinicians, into a simulated environment that replicates real-life scenarios. While traditionally clinical simulation has referred to simulation used in medical education, there is an increasing focus on its use as an investigative methodology [12]. Participants in clinical simulation studies are provided with inputs such as synthetic patient cases and clinical scenarios, which they are asked to consider while assessing the DHT in this context. This allows researchers to test DHTs in an agile and lower-cost way, while making sure that they are testing the technology with the right people, at the right time, and in the right place. The FDA has made decisions to authorize medical devices for market based in part or exclusively on novel approaches, including validation simulations, and use of synthetic data for assessment [13]. Postmarket simulation is also used to aid manufacturers to detect error patterns, validate updates to the technology, and predict future errors.

Given the iterative challenges of SaMD, and implications for regulatory approvals, we selected it as a use case for exploring clinical simulation as a research and evidence generation method. We conducted an eDelphi study to gain consensus on the research question “What criteria should be used to assess clinical simulation being used to generate evidence for SaMD?”

## Methods

### Design

**Figure 1:**
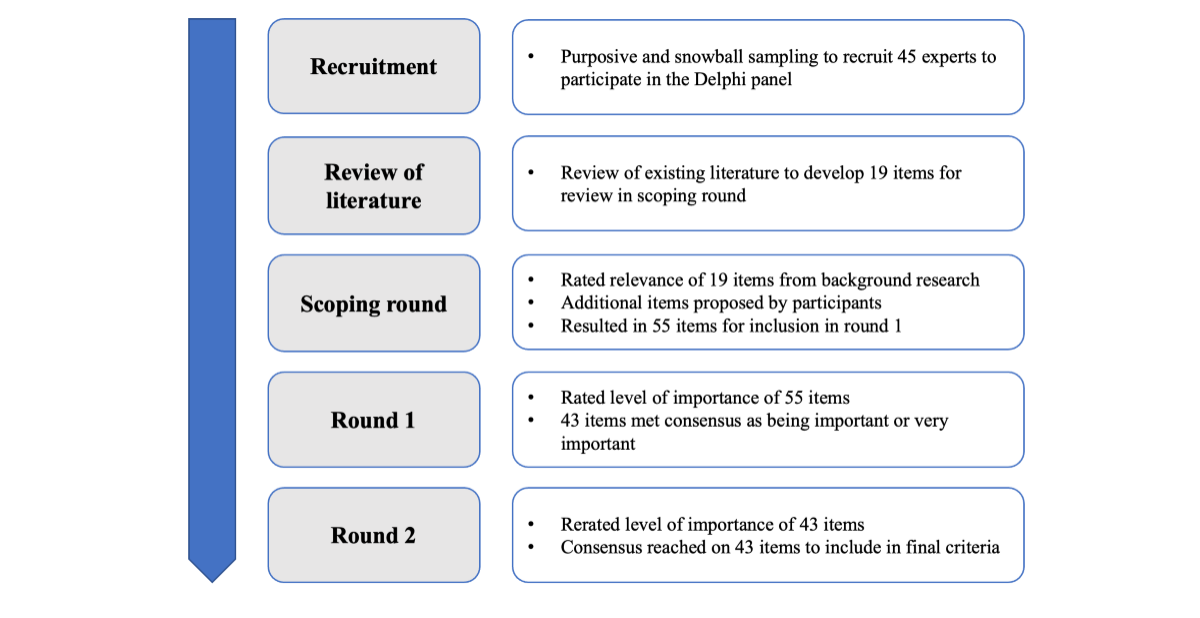
High-level overview of each stage that occurred in this Delphi study process.

The study was designed as a Delphi method using a web-based format, known as eDelphi [14], and conducted virtually between October 2022 and January 2023, in a process outlined in Figure 1. The eDelphi method can still adopt the classical Delphi method; yet, it is provided in a web-based format [15]. The Delphi method is a systematic research method that uses structured communication techniques to achieve consensus on a specific topic [15]. The participants in the process are experts who have been selected based on the research question. The Delphi process involves the participants answering several rounds of questionnaires, with an anonymized summary synthesized and shared by the Delphi facilitator between each round. Before the next round, participants are requested to review their previous answers, and the process is repeated until a predetermined stop criterion is met [14]. This allows participants to review their previous answers between rounds and consider comments provided by other participants, before responding again [15]. Given the need for criteria to guide the use of clinical simulation in evaluating SaMD, the Delphi technique is an appropriate method to build international consensus. The web-based format and asynchronous input allowed us to consult international experts [15], which was appropriate given the global applicability of the research question. The study design and reporting were in line with guidance on Conducting and REporting DElphi Studies (CREDES) [16].

### Study Participants

The study group comprised participants who were either key members in the digital health field (academia, regulatory, industry, policy makers) or experts who have been identified by the study team as having the relevant knowledge and experience to provide insights of evidence generation in the regulatory space. The term “expert” was used to define someone who has made a substantial contribution, or is a key opinion leader, in his or her relevant field, such as leading a portfolio of academic publications concerning digital health or holding a senior policy or government position in a relevant area. Study participants were reviewed with the study team to ensure that all members were satisfied with the application of the term “expert.” Study participants were selected via purposive and snowball sampling [17] to reflect a wide range of geographical locations, sectors, and experience. These sampling methods aimed to ensure that the findings were validated from technical, clinical, and system perspectives. Inclusion criteria were individuals who have expertise or experience in the area of regulation of medical devices or DHTs. Participants younger than 18 years or unable to communicate in fluent English were excluded. Participants were formally invited to participate in the study by email and received a participant information sheet and consent form. They were made aware of the aims of the research and study protocol and informed consent was obtained. Throughout the study, only those participants who completed the previous activity were invited to complete subsequent rounds. Participants were sent email reminders to complete each round.

### Scoping Activity

A literature review was conducted before the Delphi study began to identify possible criteria relevant to the use of clinical simulation in the regulation of SaMD. This informed the development of a web-based questionnaire that was piloted with several experts before formally sending it to the wider panel of study participants. This scoping activity aimed to gather information before the Delphi began and determine the initial criteria to be included in the first Delphi round [18]. Participants were sent the scoping questionnaire by email and asked to indicate whether each item identified from the literature was “relevant,” “irrelevant,” or whether they were “not sure,” with free text to provide comments for each item suggested. Items were included in the first round if >50% of the participants indicated that they were relevant. They were also asked at the end of the questionnaire whether they thought any potential criteria had been missed and should be included in the first Delphi round. They were encouraged to provide as many opinions as possible so as to maximize the chance of covering the most important opinions and issues [18]. The qualitative data were analyzed (FO) and additional items suggested by participants were included in the first round.

### Definition of Consensus

To avoid bias during the study, the definition of consensus was predefined [19]. Two criteria for consensus were applied to decide whether items should move to the next round. Items that were rated by >10% of panelists as “2: not important” or “1: not important at all” were excluded. In addition, if <60% of participants rated an item as “5: very important” or “4: important,” it was excluded. This is in line with previous Delphi studies that usually have a threshold of 60% or higher [19].

### First Round

The first Delphi round aimed to begin to build consensus on the list of items generated from the scoping activity. The list of criteria was presented to participants in a web-based questionnaire. Participants were asked to rate each item in the list on a 5-point Likert scale (“5: very important,” “4: important,” “3: neither important nor unimportant,” “2: not important,” and “1: not important at all”). Participants were also given the opportunity to provide qualitative comments, as they were asked to provide the reason behind their scores. The qualitative data were initially analyzed separately by 2 researchers, FOD and NOB, following which they discussed and finalized the themes. Thematic analysis was performed only for the first round of the Delphi to capture early comments from participants when they are likely most engaged.

### Second Round

The purpose of the second Delphi round was to build consensus on the final list of criteria for the use of clinical simulation in the regulation of SaMD. Results from the first round, along with the qualitative comments, were summarized and shared with the panelists. Participants were asked to rate each item that had progressed to the second round on the same 5-point Likert scale. The final list of items included was distinguished between different degrees of agreement—with >60% as low level of agreement, >70% as high level of agreement, and >80% as very high level of agreement. Qualitative comments provided in the second round were reviewed to provide additional context to the research team.

### Ethical Considerations

This study received ethics approval from the Science, Engineering and Technology Research Ethics Committee (SETREC) at Imperial College London (SETREC number: 22IC7862). The protocol and supporting documents were reviewed and approved by the Research Governance and Integrity Team and Head of Division/Department and did not require committee discussion as no significant ethical issues were identified. All participants were electronically provided with participant information prior and provided signed informed (electronic) consent prior to commencing the scoping round. The ability of participants to opt out at any stage of the research process was provided as part of the informed consent process. All participants and their answers provided in the study were deidentified throughout the process, including when sharing results between Delphi rounds and sharing the overall results of the study. No identifiable features of research participants were included in the manuscript. Only the research team has access to the identifiable data and they are stored securely in Imperial College London systems. Participants were not compensated for their involvement in the study.

## Results

### Initial Scoping Activity

The initial literature search yielded 19 items that were included in the questionnaire as part of the scoping activity. Thirty-nine participants from across industry (12/39, 31%), regulators (7/39, 18%), academia (15/39, 38%), and policy (4/39, 10%) completed the scoping exercise out of the 45 individuals who had initially consented (Table 1). To maintain the anonymity of the participants, it was not possible to publish more detailed information about the participants’ job roles. Participants were from 9 different countries (Table 2). There were 26 qualitative comments made in response to the final question regarding other items that had been missed, along with 132 comments made throughout the questionnaire in response to the items suggested. For each of the 19 items that were proposed to the participants, >50%, of the participants said that they were “relevant” and therefore were included in the first Delphi round. However, most criteria were rephrased or amended based on the feedback provided in the scoping activity. Overall, the scoping activity resulted in 55 items to be included in the first round, which were grouped into 7 categories: background and context, overall study design, study population, delivery of the simulation, fidelity, software and artificial intelligence (AI), and study analysis.

**Table 1:** Composition of the Delphi study participants by the sector they work in.

Sector	Recruited participants (n=45), n	Scoping activity (n=39^a^), n	First round (n=35), n	Second round (n=33), n
Industry	15	12	11	11
Regulatory	7	7	7	7
Academia	16	15	14	12
Policy	7	4	3	3

^a^One participant did not provide his or her details when submitting the web-based questionnaire and so could not be included in the participant descriptive analysis.

**Table 2:** Composition of the Delphi study participants by the country they reside in.

Country	Recruited participants (n=45), n	Scoping activity (n=39^a^), n	First round (n=35), n	Second round (n=33), n
United Kingdom	24	21	19	17
United States	6	4	4	4
Germany	4	3	3	3
Switzerland	3	3	3	3
Singapore	2	2	2	2
Australia	2	2	2	2
France	1	1	1	1
Belgium	1	1	0	0
Luxemburg	1	0	0	0
Netherlands	1	1	1	1

^a^One participant did not provide his or her details when submitting the web-based questionnaire and so could not be included in the participant descriptive analysis.

### Delphi Rounds

Following the scoping activity, 38 participants were invited to take part in the first Delphi round. One of the study participants did not provide his or her personal details when completing the scoping questionnaire and so could not be further contacted. In total, 35 participants completed the first round and 33 participants completed the second round.

#### Quantitative Analysis of the First Round

A total of 43 of the 55 items in the first round met the predefined quantitative criteria for progressing to the second round. Eleven items were excluded because >10% of the participants had rated them as “1: not important at all” or “2: not important.” However, 5 of these 11 items did not meet the second criteria for exclusion, as >60% of the participants had rated them as “very important” or “important.” One further item was excluded as less than 60% (18 participants) rated it as “5: very important” or “4: important.”

The items with the greatest proportion of participants who rated them as either “5: very important” or “4: important” included “a clear description of the SaMD being evaluated, including its purpose and intended end users” (100%), “the primary and secondary outcome measures are clearly defined, including how and when they were assessed” (100%), and “the initial orientation and any training provided to the clinicians before taking part in the clinical simulation is described” (34/35, 97% participants).

#### Qualitative Analysis of the First Round

A detailed thematic analysis was performed with the qualitative comments that were provided by the participants in the first round, where they were asked to summarize the reasons for their decisions after each section. To provide some insight, the qualitative analysis for 2 themes and their respective subthemes is discussed in the following summary.

**Figure 2:**
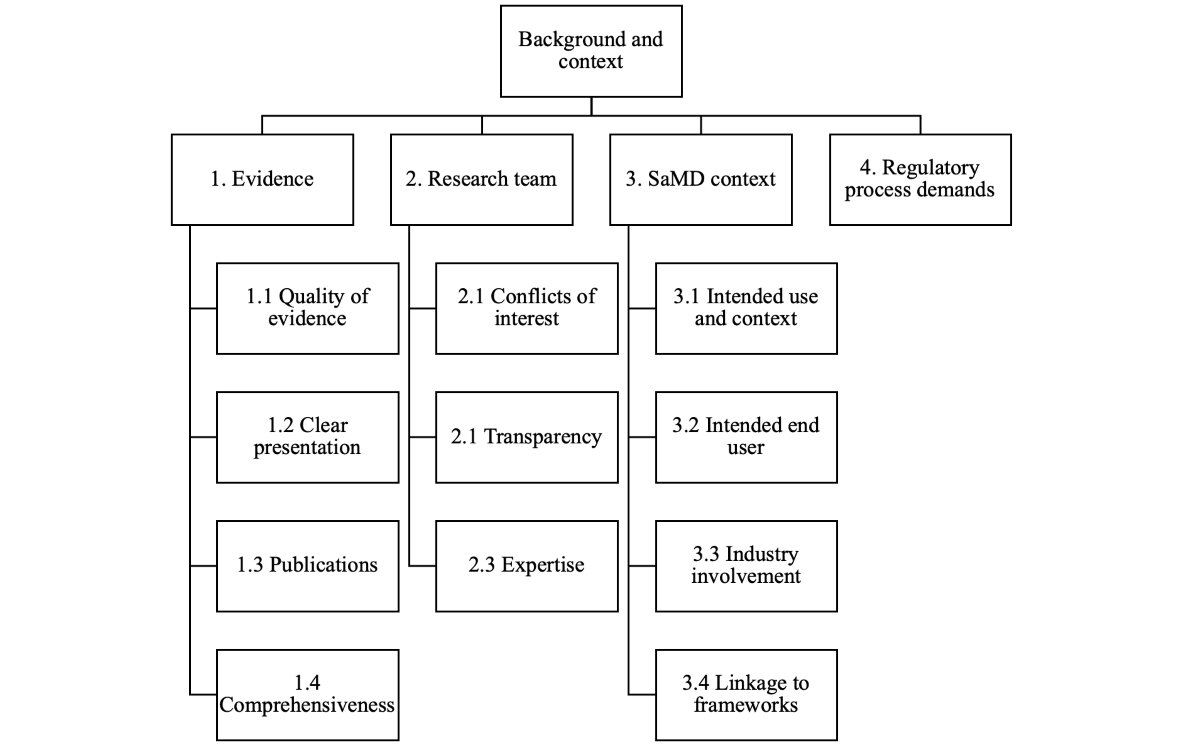
Themes and subthemes from the qualitative analysis of comments from the “Background and context” questions in the first Delphi round. SaMD: software as a medical device.

Within “background and context,” there were 4 primary subthemes: evidence, research team, SaMD context, and regulatory process demands (Figure 2). Participants felt that the “level and quality of available evidence is key” and this must be clearly presented and comprehensive in their scope. The research team’s expertise should be appropriate and transparently reported, along with any conflicts of interest. Related to the SaMD context, the intended use is “absolutely essential in evaluating the evidence generated to support a regulatory decision” along with clearly defined end users of the SaMD. The broader regulatory workload and processes should be acknowledged to ensure that work is not replicated and organizations are incentivized to adopt a clinical simulation approach to evidence generation.

**Figure 3:**
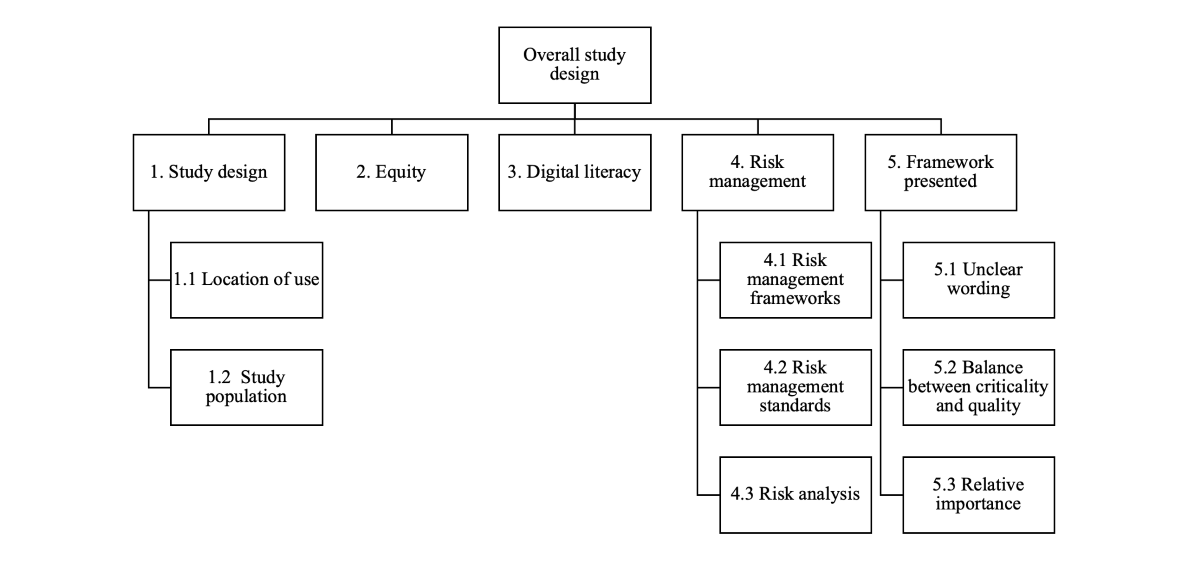
Themes and subthemes from the qualitative analysis of comments from the “Overall study design” questions in the first Delphi round.

Within “study design,” there were 5 primary subthemes—study design, equity, digital literacy, risk management, and framework presented (Figure 3). Related to study design, location of use was relevant for any SaMD products to be used in a home setting, and the study population should consider how the patient population was chosen to avoid introducing any bias or inequity. This echoes the overall importance of “understanding and eliminating reasons for inequitable access,” while another participant felt that the equity of service provision is for “decision makers not those who conduct primary research.” Risk management was discussed, and 1 participant felt that it was the “most important factor” as it impacts across many other areas, with several standards referenced by participants that should be adhered to. A broader point related to the framework that was presented in the first round is that some participants felt that there was unclear wording that may have impacted their answers.

#### Quantitative Analysis of the Second Round

All 43 of the items that met the criteria to progress to the second round also met the same criteria to be included in the final Delphi results. As a result, consensus was reached on the 43 criteria to answer the research question “What criteria should be used to assess clinical simulation being used to generate evidence for SaMD?” (Table 3). Based on these criteria, the simulation for regulation of SaMD (SIROS) framework was developed. The framework is designed to provide relevant stakeholders with clear criteria to assess clinical simulation being used to generate evidence for SaMD.

The final list of items included was also distinguished between different degrees of agreement—with >60% as low level of agreement, >70% as high level of agreement, and >80% as very high level of agreement. Five items met low level of agreement, 9 items met high level of agreement, and 29 items met very high level of agreement.

**Table 3:** Final agreed criteria in the simulation for regulation of SaMD^a^ (SIROS)^b^ framework developed from the Delphi responses and their level of consensus.

Categories and criteria		Consensus (% rating as 5: very important or 4: important), %
**Background and context**		
	Clear description of the SaMD being evaluated, including its purpose and intended end users.	100
	Description and justification of the clinical simulation performed, alongside any other research being conducted to evaluate the SaMD.	94
	Overview of the existing evidence to support the SaMD is provided.	76
	Sources of funding and other conflicts of interest are declared appropriately.	67
**Overall study design**		
	Potential limitations of the study design are discussed.	94
	Potential biases associated with the study design are discussed.	100
	Strategies to minimize potential study biases are described.	85
	Issues on equity have been considered in the overall study design, for example, high-risk patient profiles, racial disparities.	85
	Digital literacy is considered in the study design, for example, digital literacy of clinicians taking part in the clinical simulation or the digital literacy of the intended end users.	70
	Risk management in the study is described, for example, impact assessments.	76
**Study population**		
	The eligibility criteria for clinicians who took part in the clinical simulation are representative of the intended end users, for example, staff level, qualification, experience.	91
	The sampling and recruitment methods used to recruit clinicians who took part in the clinical simulation are clearly described.	70
	The number of clinicians who took part in the clinical simulation is provided.	85
	Issues on equity were considered in the sampling and recruitment process to ensure representativeness.	79
**Delivery of the simulation**		
	The environment in which the clinical simulation took place is described, for example, physical or virtual location, type of health care facility.	85
	The timing of the clinical simulation is described, for example, time of day, length of time taken.	67
	The equipment used for the clinical simulation is described.	88
	The facilitator (the individual who facilitated the clinical simulation for the clinicians), if any, is described, for example, what role they took, how many there were, what input they had.	64
	The initial orientation and any training provided to the clinicians before taking part in the clinical simulation are described.	88
	When the clinical simulation was being performed, the SaMD was described in sufficient detail to the clinicians taking part in the clinical simulation to allow them to evaluate it.	88
**Fidelity**		
	There is a clear analysis, considering the risk and impact, of the different levels of fidelity, for example, high, medium, and low, required for various aspects of the clinical simulation.	73
	A lack of fidelity in any aspect of the clinical simulation is explained and justified, for example, fidelity in one aspect of the scenario may not be required for the SaMD being assessed.	73
	The clinical simulation has high conceptual fidelity that meets the intended use of the SaMD.	88
	The clinical simulation uses high-fidelity synthetic patient cases that meet the intended use of the SaMD.	88
	The clinical simulation has high clinical scenario fidelity that meets the intended use of the SaMD.	94
	The clinical simulation has high health care facilities fidelity that meets the intended use of the SaMD.	73
	The methodology and rationale for developing the synthetic patient cases are described.	82
	The overall representativeness of the synthetic patient cases is described.	88
	Potential limitations of the synthetic patient cases are discussed.	91
	Potential data bias in development of the synthetic patient cases is discussed.	82
	Strategies to minimize potential data bias associated with synthetic patient cases are discussed.	79
**Software and AI^c^**		
	Any continuous machine learning algorithms embedded in the SaMD are described.	100
	The design and development of any continuous machine learning algorithms embedded in the SaMD are described.	82
	Any continuous machine learning algorithms are reviewed at regular intervals to monitor their changes from the initial setup.	97
	Any software updates to the SaMD made since the clinical simulation study are described and justified.	94
**Study analysis**		
	The primary and secondary outcome measures are clearly defined, including how and when they were assessed.	97
	Rationale and justification for the chosen primary and secondary outcome measures are provided.	94
	The usability of the SaMD is assessed as part of the clinical simulation.	79
	The feasibility of the SaMD is assessed as part of the clinical simulation.	75
	The impacts of any unintended consequences, for example, harm or clinical risk from the study are described.	88
	The data analysis performed is clearly described, for example, statistical methods and the unit of analysis used (eg, individual, team, group).	100
	The generalizability of the study findings is discussed, for example, to other populations or clinical scenarios.	88
	Sensitivity analysis is performed to assess the robustness of the clinical simulation findings.	88

^a^SaMD: software as a medical device.

^b^SIROS: simulation for regulation of SaMD.

^c^AI: artificial intelligence.

## Discussion

### Principal Findings

Background and context criteria viewed as most important to use in clinical simulation to generate evidence for SaMD were a clear description of the SaMD being evaluated, including purpose and intended users, and description and justification of the simulation performed; this was alongside any other research being conducted to evaluate the SaMD. While remaining important, the criteria for appropriately declaring sources of funding and other conflicts of interest scored less highly. In terms of overall study design criteria, the participants viewed it as important that potential limitations of study design and potential biases associated are discussed. Similarly, strategies to minimize potential study biases being discussed, particularly regarding issues of equity (eg, high-risk patient profiles, racial disparities), were also noted as important. Again, while consensus was reached to include information on how digital literacy is considered in the study design, the participants did not rate this as highly as the other areas. The study population criteria rated as most important included the eligibility criteria for clinicians who took part in the clinical simulation being representative of the intended end users (eg, staff level, qualification, experience) and transparency around the number of clinicians who took part in the simulation.

The highest rated delivery of the simulation criteria included the need to describe the environment in which the simulation took place (eg, physical or virtual and type of facility), the equipment used, the initial orientation and training provided to clinicians before taking part, and a description of how the SaMD was described to clinicians before taking part. The fidelity criteria rated as most important were that the clinical simulation has high conceptual fidelity that meets the intended use of the SaMD, it uses high-fidelity synthetic patient cases, and the simulation has high clinical scenario fidelity. Beyond these elements, the participants highly rated the need to describe the methodology and rationale for developing the synthetic patient cases, the overall representativeness of the synthetic patient cases, their potential limitations, and possible data biases in their development. On software and AI, the participants viewed all criteria as highly important, including the need to describe any continuous machine learning (ML) algorithms embedded in the SaMD, including their design and development; ensure that they are reviewed at regular intervals to monitor their changes; and describe and justify any software updates to the SaMD since the clinical simulation study.

The most important study analysis criteria included the need to clearly define primary and secondary outcome measures, and provide a rationale and justification for selecting these, the impacts of any unintended consequences (eg, harm) from the study, data analysis methods, generalizability of the findings, and results of a sensitivity analysis to assess the robustness of the clinical simulation findings.

The inclusion of the majority (43/55, 78%) of criteria presented to the Delphi participants suggests that there are substantial data collection and reporting that need to be considered and executed if clinical simulation is to be increasingly used to authorize medical devices for market based in part or exclusively on validation simulations. However, the benefits of such evidence generation and reporting are also clear; evidence can be used to achieve regulatory authorization and, postmarket, can detect error and validate technologies [13]. Developing the SIROS framework with guidance across the 43 criteria presented in this study can enable manufacturers to work methodically on evidence generation and regulatory submissions for more streamlined SaMD approval. A next step will be to develop an accompanying checklist that makes the SIROS framework actionable for SaMD developers.

While our study is the first of its kind in gathering consensus on how to assess clinical simulation being used to generate evidence on SaMD, the participants in our research viewed many of the criteria as important. This is complementary to findings from other contemporary studies on methods to generate evidence on DHTs. More generally, the participant comments on the importance of such guidance are in line with research by Day et al [11], who note that many digital health start-ups have limited clinical robustness, as measured by regulatory filings and clinical trials. The authors note a lack of meaningful clinical validation for almost half of digital health companies (44% had a clinical robustness score of 0), highlighting a lack of guidance such as the SIROS framework. In recent years, national guidance and regulations have been developed to enable rapid assessment of DHTs. In the United States, the Digital Medicine Society has created a regulatory compass tool titled RegPath, with input from the FDA, to enable improved understanding of whether a specific DHT falls within FDA regulation, and if so, which regulatory pathway is relevant [20]. In Europe, German regulators have developed a fast-track pathway for digital health applications (in German, DiGA) to be reimbursed by statutory health insurances [21,22], a model that will now be employed in other European countries. The importance of developing a framework for assessing clinical simulation can therefore be seen as a next step to complement ongoing regulatory developments internationally, where there are limited tailored guidelines or frameworks at present.

The 7 areas and associated criteria agreed by the participants are in line with existing literature and research studies that used clinical simulation to evaluate DHTs. Gardener et al [23] used clinical simulation to evaluate a clinical decision support tool for matching patients with cancer to clinical trials. Participants in the research stated that they were provided sufficient guidance on the exercises and enough clinical information in the synthetic patient cases, though a small number noted that they would have preferred more information on histology information. Such findings suggest the importance of providing regulators with information on initial orientation and training provided to clinicians before taking part, as a key factor in the clinical simulation’s success. Gardener et al [23] reported that the participants noted a lack of familiarity with the novel solution that could potentially challenge the clinical simulation approach. However, as there are few published studies on the role of clinical simulation to evaluate DHTs, there is an urgent need for further research in this area that can not only use the areas developed through our research but also validate these areas by applying them in practice. Similarly, there is the potential for the 7-dimension SIROS framework developed to be used in the evaluation of other types of DHTs where many similar issues will be of concern to regulators.

Echoing the well-researched importance of fidelity of simulation used in health education [24,25], participants in the Delphi identified this as a key area where researchers were required to outline how the clinical simulation sought high fidelity with planned future use of the SaMD. The specific context in which the SaMD is intended to be used must be considered in planning for the clinical simulation to enable accurate reporting, with particular attention paid to high-fidelity synthetic patient cases, and their implications for representativeness and equity. In this regard, the evidence required is similar to that of all DHTs developed with AI or ML methods. For example, the Good Machine Learning Practice for Medical Device Development: Guiding Principles developed by the FDA, UK Medicines and Healthcare products Regulatory Agency (MHRA), and Health Canada in 2021 encourage good practice in medical device development using AI or ML, including the reduction of bias through representative clinical study participants and data sets [26]. In cases in which the SaMD being simulated uses continuous self-learning algorithms, the Delphi participants highlighted the need for reporting of plans for continuous monitoring and other steps to maintain quality and safety as part of defining, controlling, and improving software life cycle processes outlined in ISO/IEC (International Organization for Standardization/International Electrotechnical Commission) 12207 [27] and adhering to relevant local legislation and regulatory guidance. Such views were echoed by Carolan et al [6], who note the need for international standards and guiding principles addressing the uniqueness of SaMD with a continuous learning algorithm.

The importance of presenting issues of bias and equity through the simulation process is a central element of how to assess clinical simulation being used to generate evidence on SaMD according to our research participants. This is perhaps unsurprising given the increasing research outlining the potential risk of bias and increased health inequities associated with poorly developed or implemented DHTs, including AI or ML [7,28-30]. Guo et al [31] identified a range of relevant tools and frameworks providing guidance on different aspects of bias in evidence generation studies; these include Quality in Prognostic Studies (QUIPS), Cochrane risk-of-bias tool for randomized trials (RoB2), PROBAST: A Tool to Assess the Risk of Bias and Applicability of Prediction Model Studies, and the Risk Of Bias In Nonrandomized Studies - of Interventions (ROBINS-I). Such frameworks offer SaMD developers a ready source of information to address and report on issues related to bias as part of clinical simulation research during submissions for regulatory approval. In response to the challenge of adaptive technologies, the proposed FDA framework for modifications to AI- or ML-based SaMD further seeks to ensure that safety and effectiveness are maintained [32].

Current tools and guidance remain at best a stopgap until regulatory environments and international guidelines can be developed to ensure that SaMD is developed and deployed with a clearer understanding on its impact on quality and safety through the software life cycle. To advance this process, manufacturers should engage with regulators and propose clinical simulation methods for the purpose of regulatory approval. While there are a few existing examples of clinical simulation data in part or wholly being used as evidence for regulatory approvals, developing more real-life use cases will enable the development of best practice guidelines and lessons learned that will benefit all stakeholders. Regulators and notified bodies should also work with manufacturers on the application of clinical simulation. Providing greater clarity on what they would like to see from data and how they can best be collected and presented for SaMD approval will enable manufacturers to be increasingly targeted in their approach to clinical simulation.

### Limitations

Delphi studies traditionally begin with an open-ended question for participants to provide numerous open-ended responses. The results of this initial ideas generation stage are then analyzed, summarized, and presented in subsequent rounds [18]. However, some studies have chosen a different approach, where preexisting information is initially described to participants and on which they are asked their opinion [33,34]. This approach was taken in this study as outlined in the procedure. This can be justified as it aims to prepare participants for the upcoming rounds and can reduce the potentially overwhelming task of data analysis. However, limitations regarding potential bias of responses exist along with exclusion of relevant ideas that participants may have contributed if they were requested in an open-ended format.

There was some confusion apparent in the panelists’ qualitative comments, particularly in the scoping round, about terminology used and the research question context. For example, many panelists misinterpreted the terms “study participants,” “intervention,” and “evaluation opinion” most commonly. This may have been due to the complexity of the use case scenario and with no practical scenarios provided to help with its understanding. To overcome this, an analysis of the comments regarding lack of clarity was carried out after the scoping activity and used to improve the wording of the questions and criteria for the first round.

Despite efforts to recruit a global cohort that is representative across high-, low-, and middle-income countries, most participants came from high-income countries, particularly the United Kingdom. This may have arisen due to several factors, such as the informal networks from the study sponsor being predominantly based in the United Kingdom, or that the global SaMD community has a greater base in high-income countries. This may have introduced bias to the results, with answers representing the views of UK-based participants and their country-specific setting, as opposed to gaining a more diverse range of global responses as desired. Regardless, further research is required to ensure that the study results are applicable to other settings and in other country contexts.

The 2 predefined criteria for deciding on whether to include criteria between successive rounds may have led to some potentially important items being removed unnecessarily. As mentioned previously, in the quantitative analysis of the rounds, any item was removed if it was rated by more than 10% of panelists as “not important” or “not important at all” or if less than 60% of panelists rated that it was “important” or “very important.” The analysis of the first round led to 5 items that met the former criteria but did not meet the latter. However, given that it did not meet one of the predetermined criteria it was excluded from the second round. Therefore, these 5 items that did not progress to the second round may have included important information for regulators to consider when evaluating clinical simulation methods but were excluded.

### Conclusions

The Delphi exercise undertaken enabled the development of the SIROS framework of 7 domains and associated criteria for assessing clinical simulation being used to generate evidence on SaMD. Implementation of the criteria generated can enable faster uptake of high-potential technologies. The framework is highly relevant in the current health regulatory landscape where there is limited guidance or regulatory oversight on SaMD implementation or use across the life cycle of the technology. Participants in the Delphi study identified key areas of concern, specifically around the importance of fidelity of simulation, and its reporting, as well as the challenge of bias in SaMD which risks a reliance on ML algorithms trained on inadequate data sets. These aspects, as well as the other areas and criteria agreed through Delphi consensus, must be addressed by both developers and regulators as regulatory requirements for SaMD advancement in the coming years.

## Abbreviations

AI: artificial intelligence
CREDES: Conducting and REporting DElphi Studies
DHT: digital health technology
FDA: Food and Drug Administration
ISO/IEC: International Organization for Standardization/International Electrotechnical Commission
MHRA: Medicines and Healthcare products Regulatory Agency
ML: machine learning
QUIPS: Quality in Prognostic Studies
RoB2: Cochrane risk-of-bias tool for randomized trials
ROBINS-I: Risk Of Bias In Nonrandomized Studies - of Interventions
SaMD: software as a medical device
SETREC: Science, Engineering and Technology Research Ethics Committee
SIROS: simulation for regulation of software as a medical device

## Data Availability

The data sets generated during this study are available from the corresponding author on reasonable request.
